# Identifying Children with Developmental Coordination Disorder via Parental Questionnaires. Spanish Reference Norms for the DCDDaily-Q-ES and Correlation with the DCDQ-ES

**DOI:** 10.3390/ijerph17020555

**Published:** 2020-01-15

**Authors:** Rebeca Montes-Montes, Laura Delgado-Lobete, Javier Pereira, Marina M. Schoemaker, Sergio Santos-del-Riego, Thais Pousada

**Affiliations:** 1 Centre for Information and Communications Technology Research (CITIC), TALIONIS Research Group, University of A Coruña, 15008 Coruña, Spain; rebeca.montes@udc.es (R.M.-M.); javier.pereira@udc.es (J.P.); thais.pousada.garcia@udc.es (T.P.); 2 Faculty of Health Sciences, Health Integration and Promotion Research Unit (INTEGRA SAÚDE), University of A Coruña, 15011 Coruña, Spain; sergio.santos.delriego@udc.es; 3 Center for Human Movement Sciences, University Medical Center Groningen, University of Groningen, 9713 AV Groningen, The Netherlands; m.m.schoemaker@umcg.nl

**Keywords:** developmental coordination disorder, assessment, DDCDaily-Q, DCDQ, activities of daily living, daily participation, daily performance, occupational therapy

## Abstract

Developmental Coordination Disorder (DCD) is one of the most prevalent neurodevelopmental disorders in school-aged children, with major consequences in psychosocial and physical health. Adequate identification of this disorder is necessary to prompt effective interventions. The aims of this study were to develop the Spanish adjusted reference norms for the DCDDaily-Q and to test the correlation and agreement between the Spanish versions of the DCDDaily-Q (DCDDaily-Q-ES) and the Developmental Coordination Disorder Questionnaire (DCDQ-ES), two reliable instruments to assess motor performance and DCD. Clinically relevant percentiles were calculated for the DCDDaily-Q-ES using a representative sample of Spanish children aged 5 to 10 years (*n* = 356; M = 7.3 years, SD = 1.8; boys = 50%). Pearson’s correlation coefficient and intraclass correlation coefficient (ICC) were used to determine correlation and agreement between questionnaires, respectively. A moderate and significant correlation and agreement between DCDDaily-Q-ES and DCDQ-ES was found (*r* = 0.406; ICC = 0.381; *p* < 0.001). Differences in daily participation and performance were found between age groups, but not between boys and girls. Spanish age-adjusted percentiles and cutoff scores for DCDDaily-Q-ES are provided. This study offers further validation and relevant information regarding assessment of DCD and has practical implications for clinical practice and research.

## 1. Introduction

Developmental Coordination Disorder (DCD) is one of the most prevalent neurodevelopmental disorders in school-aged children, and it is considered one of the major health concerns in this population [1,2,3,4]. Consequences of DCD often span across psychosocial, occupational, and physical health dimensions, and usually persist into adulthood [2,5,6,7].

In addition to motor coordination difficulties, children and adolescents with DCD have an increased risk for mental and psychosocial health issues, such as depression and anxiety and other internalizing problems [8,9,10,11], problems with social interactions and victimization [2,12,13,14], emotional and behavioral issues [11,13], and lower self-worth and self-esteem than children without motor coordination difficulties [15]. Regarding physical problems, children with DCD are at higher risk for obesity, higher waist circumference and body fat percentage [16,17], poorer cardiorespiratory fitness, and lower flexibility, muscle strength, and muscle endurance [6,18,19,20]. Overall, children and adolescents with DCD suffer lower health-related quality of life than their typically developing peers [14,15,21].

Despite this disorder being highly underdiagnosed in Spain, a recent study suggests that 12% of Spanish children aged 6 to 12 years have probable DCD [22,23]. To get a diagnosis of DCD, the child must present a motor deficit (criterion A) that affects performance during activities of daily living (ADL) (criterion B). This motor deficit must be present since early development (criterion C) and cannot be better explained by other medical conditions (criterion D) [1]. Early detection and identification are recommended as they lead to prompt intervention and guide efforts and resources towards a reliable and definite diagnosis. The Developmental Coordination Disorder Questionnaire (DCDQ) [24] is recommended to evaluate criterion B [2] and it has been recently cross-culturally adapted and preliminarily validated in Spanish children [25]. Even though the DCDQ addresses motor performance during ADL, a comprehensive evaluation of self-care activities is lacking [26].

Self-care and self-maintenance activities should be systematically assessed as self-care participation and performance are severely affected in children with DCD [27,28,29,30,31]. Some studies have reported that DCD has a greater impact on self-care functioning than on gross motor or fine motor performance [27,28]. Moreover, both children with DCD and parents of children with DCD perceive difficulties in self-care functioning as one of their main concerns [14,32,33]. Specific self-care activities that children with DCD struggle with include dressing (managing buttons and zippers, orientating and manipulating socks, tying shoelaces), eating (using a knife and fork, pouring liquids, opening wrapper/package, noticing quantity of food in mouth or fork), and toileting (drying oneself after bathing or showering, brushing teeth or hair, managing toothpaste, wiping oneself clean) [27,29,30,31].

The DCDDaily-Q [34] is a newly developed instrument to comprehensively assess ADL, especially focusing on self-care, fine motor and gross motor performance, and participation. Research suggests that it has an extraordinary discriminant capacity and it can effectively identify children at risk of DCD, but more studies are needed [2,34]. The DCDDaily-Q was recently cross-culturally adapted and psychometrically validated in Spain, showing an excellent construct validity and discriminant capacity to identify children with neurodevelopmental disorders (to be published). However, due to limitations in sample size, reference norms for Spanish children were not developed, and its concurrent validity with the Spanish version of the DCDQ (DCDQ-ES) has yet to be tested. Previous research shows that population-adjusted reference norms should always be operated when assessing motor performance [35,36,37]. To date, the original Dutch cutoffs are the only available criteria to identify children at risk of DCD when using the DCDDaily-Q, so it is unknown if these cutoffs are also suitable for children within different contexts or regions.

The aims of this study are (1) to develop the Spanish adjusted reference norms for the DCDDaily-Q using a representative sample of Spanish children, and (2) to test the correlation and agreement between the DCDDaily-Q-ES and the DCDQ-ES in Spanish context.

## 2. Materials and Methods

### 2.1. Participants and Procedures

Data from 356 children aged 5 to 10 years were collected between January and December 2019. Participants came from 17 randomly selected mainstream schools located in eight geographic locations in northwest, north, and central Spain. Geographic location selection was made by convenience and considering different sociodemographic factors, such as rural or urban settings and family educational background. None of the participants had an existing or previous diagnosis of any learning or developmental disorder as reported by schools and parents.

The DCDDaily-Q-ES was sent to the parents via school intermediation, and returned to the researchers after completion. Additionally, 266 parents also completed the DCDQ-ES. This study was carried out following the rules of the Declaration of Helsinki and it was approved by the Research Ethics Committee of Galicia in Spain (code 2018-606, date of approval: December 2018). Data were collected anonymously. All participants consented to take part in the study anonymously and confidentially.

### 2.2. Measurements

#### 2.2.1. Spanish Version of the DCDDaily-Q

The DCDDaily-Q is a 23-item parental questionnaire that addresses children’s participation and performance in a broad range of ADL, including self-care (10 items), fine motor (7 items), and gross motor activities (6 items) [34]. Parents are asked to rate their children’s performance for each item in comparison to a given description of what is considered the right way to perform the activity (1 = good, 2 = medium, 3 = poor). The DCDDaily-Q total performance score ranges from 23 to 69, where higher scores indicate poorer performance. Apart from performance, the DCDDaily-Q also evaluates participation in ADL on a four-point scale (1 = regularly, 2 = sometimes, 3 = seldom, 4 = not yet/never). The total participation score ranges from 23 to 92, and higher scores indicate less participation.

The DCDDaily-Q has excellent psychometric properties and discriminant capacity to identify children with DCD (Cronbach alfa = 0.85; sensitivity = 88%; specificity = 92%) [34]. The Spanish cross-cultural adaptation (DCDDaily-Q-ES) was successfully undertaken following the international guidelines [38,39] and shows excellent fit to the three-factors structure and good reliability (Cronbach alfa = 0.82).

#### 2.2.2. Spanish Version of the DCDQ

The DCDQ was designed to identify motor problems and probable DCD in 5- to 15-year-old children [24], and it is the most used measurement to operationalize criterion B of the diagnostic criteria for DCD [2]. The DCDQ is a parental questionnaire consisting of 15 items covering three subscales: Control during movement (6 items), fine motor/handwriting (4 items), and general coordination (5 items). Parents are asked to rate their children’s performance on a five-point scale. The DCDQ total score ranges from 15 to 75, where higher scores indicate better performance.

The DCDQ shows good psychometric properties (Cronbach alfa = 0.94; overall sensitivity = 85%; overall specificity = 71%) [24]. This instrument has been successfully cross-culturally adapted to European Spanish (DCDQ-ES), and its psychometric validation proved that it is a reliable and valid measure to assess motor coordination in Spanish children (Cronbach alfa = 0.86) [25].

### 2.3. Data Analysis

Statistical analysis was performed with SPSS version 20.0 (SPSS Inc., Chicago, IL, USA). A value of *p* < 0.05 was considered statistically significant. One-way ANOVA analyses were conducted to calculate the effect of age on the DCDDaily-Q-ES participation and performance total and subscale scores. Bonferroni post hoc tests were used to examine differences between age groups. An independent samples *t*-test was conducted to analyze sex differences in DCDDaily-Q-ES participation and performance total and subscale scores.

Several percentiles for participation and performance total and subscales were calculated. The 85th and 95th percentiles are usually recommended to serve as cutoffs to determine the presence of DCD in clinical practice and research, respectively [40,41]. As additional percentiles may be useful for clinical practice and research, the 80th, 90th, and 96–99th percentiles were also considered.

The correlation between the DCDDaily-Q-ES performance total and subscale scores and the DCDQ-ES total and subscale scores was determined calculating Pearson correlation coefficients. The intraclass correlation coefficient (ICC) was used to determine agreement between the DCDDaily-Q-ES performance scale and the DCDQ-ES. ICC estimates and their 95% confidence intervals were calculated based on a one-way random model. In order to determine agreement between the DCDDaily-Q-ES and DCDQ-ES using ICC, the DCDQ-ES total scale score was recalculated reversing DCDQ-ES item scores from 5 (worst performance) to 1 (better performance) so that both DCDDaily-Q-ES performance and DCDQ-ES scale scores went in the same direction. Raw DCDDaily-Q-ES performance and reversed DCDQ-ES total scores were then transformed to the 0–100 range to enable ICC calculation and estimation of differences between both measurements.

## 3. Results

Sociodemographic characteristics of the sample are shown in Table 1. All age groups were balanced by sex (boys and girls = 50.0% in each group).

Using the Bonferroni post hoc test, ANOVA showed no differences in general participation or performance between children aged 7 or 8 years and children aged 9 or 10 years as measured by the DCDDaily-Q-ES (Table 2). Children aged 7 years and older scored significantly lower than children aged 5 or 6 years in self-care, fine motor, and gross motor participation and performance subscales of the DCDDaily-Q-ES. According to their parents, boys participated more often in gross motor ADL than girls, and girls performed fine motor ADL significantly better than boys, but overall, there were no sex differences in total performance or participation scores (Table 3). Therefore, percentiles for all subscales, total participation, and total performance scales of the DCDDaily-Q-ES were calculated separately for children aged 5 to 6 years and for children aged 7 years and older (Table 4).

Interpretation guidelines for the Spanish percentile cutoffs of the DCDDaily-Q-ES are shown in Appendix A Table A1. These recommendations have been developed using the original Dutch DCDDaily-Q manual as a guideline to facilitate communication when reporting DCDDaily-Q results across contexts and studies.

Overall, moderate and significant correlations were found between the DCDDaily-Q-ES performance total and subscale scores and the DCDQ-ES total and subscale scores (Table 5). The highest correlations were found between DCDDaily-Q-ES performance total and DCDQ-ES total scales (*r* = 0.406, *p* < 0.001), fine motor ADL and fine motor/handwriting scales (*r* = 0.359, *p* < 0.001), and self-care ADL and DCDQ-ES total scales (*r* = 0.356, *p* < 0.001). Correlation between the DCDDaily-Q-ES and DCDQ-ES was higher in children aged 7 years and older than in younger children (*r* = 0.509, *p* < 0.001 vs. *r* = 0.273, *p* < 0.01 respectively).

Agreement between both measurements in the overall sample was poor to moderate and significant (ICC = 0.381, 95% CI = 0.273–0.479, *p* < 0.001), and moderate and significant in children aged 7 years and older (ICC = 0.489, 95% CI = 0.366–0.595, *p* < 0.001), but poor and nonsignificant in children aged 5 and 6 years (ICC = 0.154, 95% CI = −0.048–0.343, *p* = 0.067). Differences in DCDDaily-Q-ES performance scale and DCDQ-ES total scale were higher in younger children (*t*(148.565) = 4.919, *p* < 0.001).

## 4. Discussion

This is the first study to develop reference norms for the DCDDaily-Q in new cultural contexts. Findings support the need to develop population-adjusted percentiles and cutoffs when assessing motor performance and daily participation. Correlations and agreement between the Spanish versions of the DCDDaily-Q and the DCDQ were moderate overall, but stronger in children aged 7 years and older.

According to parents, children younger than 7 years participated less and performed poorer than older children in all DCDDaily-Q-ES scales, which is consistent with previous research [34]. It is to be expected that motor performance and participation improve with age, as children get more proficient with experience, especially in self-care ADL, and therefore, they can engage in a wider range of activities as they grow older [23,31].

No overall differences in daily participation or performance were found between boys and girls. Results regarding the influence of sex on motor performance are inconclusive, as some studies have found differences while others have not [23,34,42,43]. In this study, girls were reported to outperform boys in fine motor activities, but to participate less in gross motor activities, which is an often-described outcome in literature [23,44,45,46,47,48].

The Spanish cutoff scores belonging to the 85th and 95th percentiles are higher than Dutch ones, indicating an overall less frequent participation and poorer performance of ADL in Spanish children [34]. Sample distribution may help explain this difference, as the Spanish sample is larger and better sex-balanced in each age group, but cultural factors are also to be considered. Findings from previous studies suggest differences in patterns of motor performance between children from Southern, Central, and Northern Europe, with better scores in northern regions, and thus making it necessary to develop country-specific cutoffs for the Movement Assessment Battery for Children—Second Edition (M-ABC2) or DCDQ [35,37,49,50,51,52]. Differences in scores belonging to the 85th and 95th percentiles of the DCDDaily-Q between Spanish and Dutch children were between two and five points, demonstrating the need to develop specific cutoffs for each population and context, as evaluating the risk for DCD in Spanish children using Dutch criteria would lead to false-positive outcomes and inaccurate diagnoses.

The authors recommend considering the 85th percentile of the performance scale as indicative of DCD for criterion B in a clinical context. Conversely, the 95th percentile should be considered in research works, especially in population-based studies [40,41]. It is necessary to emphasize that a definite diagnosis of DCD should only be made after assessment of all four criteria by a multidisciplinary team, which should always include an occupational therapist.

As described in previous research, a moderate but significant correlation between the DCDDaily-Q total performance score and the DCDQ total score was found [34], which further shows the concurrent validity of both measurements in a Spanish context. Several reasons explain why a perfect correlation or agreement between the DCDDaily-Q and the DCDQ is not to be expected. Firstly, although both questionnaires assess motor performance during ADL, different types of ADL are evaluated in each measurement. The DCDQ focuses on activities related to control during movement, fine motor skills, and general coordination, and includes just one item that specifically addresses self-care performance. In contrast, the DCDDaily-Q primarily evaluates self-care ADL (43.5%), apart from fine motor (30.4%) and gross motor activities (26.1%).

Secondly, parents are required to make slightly different assessments in each questionnaire. When using the DCDQ, parents are asked to compare the degree of coordination that their child has with other children of the same age, whereas the DCDDaily-Q offers a description of what is considered the right way to perform each of the 23 activities so that parents can determine how well their child does the activity.

Finally, age was a relevant factor, as correlation and agreement between DCDDaily-Q-ES and DCDQ-ES were higher in children aged 7 to 10 years. Furthermore, children aged 5 and 6 years showed higher variance in motor ADL performance than older children, especially in self-care ADL, and differences between both measurements were also greater in this age group, suggesting larger variability in motor performance during early development. This is consistent with previous research and adds to the evidence for not making a definite diagnosis of DCD in very young children [2]. Based on these findings, it is highly recommended to assess criterion B, the impact of the motor deficit on daily life, with both the DCDDaily-Q and the DCDQ, especially in children aged 5 or 6 years, so that the clinical team can gather more comprehensive information regarding daily functioning.

This study is subject to some limitations and future research directions. It was not possible to calculate sensitivity and specificity of the Spanish 85th and 95th percentiles to identify children with a formal diagnosis of DCD, as DCD is a highly unknown disorder in Spain, and most Spanish children with motor coordination difficulties go unnoticed in medical evaluations [22,23]. Having two culturally adapted, valid, reliable, and accessible instruments to assess the presence of DCD may increase awareness of DCD in Spain. The sample came from three specific locations in Spain, but it included children from different settings and social backgrounds. The presented sample was larger and more balanced by sex than the Dutch sample, but additional efforts should be made in future studies to gather larger samples to test these findings in other countries or cultural contexts. Future research should further examine potential differences in DCDDaily-Q outcomes in children from different countries. Daily participation is not only restricted in children with DCD, but also in children with other relatively common neurodevelopmental disorders, such as Attention Deficit and Hyperactivity Disorder (ADHD) and Autism Spectrum Disorders [53,54], and therefore, the DCDDaily-Q could be an effective measurement to assess participation issues in several neurodevelopmental difficulties. As most children with a diagnosis of ADHD encounter motor coordination problems as well [2,55], future research should validate whether this questionnaire can offer relevant information about motor performance of children with ADHD in specific occupational areas. The DCDDaily-Q-ES and the DCDQ-ES are suitable and accurate instruments to use in future studies aiming to identify DCD in children or to explore motor performance and participation.

## 5. Conclusions

Limitations in ADL participation and performance are core factors in DCD, and they must be adequately assessed when addressing criterion B for DCD diagnosis. The current study provides percentiles for motor performance and participation in several daily living areas and cutoff scores to identify 5- to 10-year-old children at risk of DCD in Spain using a large, representative sample of typically developing children. The DCDDaily-Q-ES and DCDQ-ES have moderate correlation and agreement, which strengthens their concurrent validity.

This study has important implications for both the clinical context and research. Health practitioners and rehabilitation professionals, such as physical and occupational therapists, can use two reliable, valid, user-friendly, and freely available tools to assess motor performance during ADL and criterion B of the diagnostic criteria for DCD. Additionally, these findings may contribute to enhance research and clinical exploration of DCD in Spain in order to improve awareness of this disorder in the Spanish population.

## Figures and Tables

**Table 1:** Sociodemographic characteristics of the sample (*n* = 356).

Sociodemographic Factors	Participants	Mean (SD) or %
Age (in years)	356	7.3 (1.8)
Age group 1 (5–6 years)	136	38.2
Age group 2 (7–8 years)	106	29.8
Age group 3 (9–10 years)	114	32.0
Boys	178	50.0
Girls	178	50.0
Northwest Spain	191	53.7
North Spain	147	41.3
Central Spain	18	5.1
Urban setting	281	78.9
Rural setting	75	21.1
High family educational level (university studies)	182	57.1
Medium-low family educational level (nonuniversity studies)	137	42.9

**Table 2:** Differences in DCDDaily-Q-ES subscales and total scores for participation and performance in activities of daily living (ADL) across age groups (*n* = 356).

DCDDaily-Q-ES Subscales	Age Group 1 *n* = 136	Age Group 2 *n* = 106	Age Group 3 *n* = 114	
	Mean (SD)	Mean (SD)	Mean (SD)	*p* Value within Groups
Participation				
Self-care ADL	16.8 (3.8)	13.9 (2.6)	13.3 (2.5)	<0.001 ^a^; <0.001 ^b^; 0.389 ^c^
Fine motor ADL	9.8 (2.6)	9.0 (2.0)	9.0 (2.2)	0.015 ^a^; 0.021 ^b^; 1.00 ^c^
Gross motor ADL	12.8 (2.8)	11.6 (2.8)	12.5 (3.0)	0.004 ^a^; 0.954 ^b^; 0.086 ^c^
Total ADL	39.5 (7.4)	34.5 (5.9)	34.8 (6.1)	<0.001 ^a^; <0.001 ^b^; 1.00 ^c^
Performance				
Self-care ADL	14.9 (3.1)	12.7 (2.5)	11.8 (1.7)	<0.001 ^a^; <0.001 ^b^; 0.023 ^c^
Fine motor ADL	9.8 (2.3)	8.6 (2.0)	8.0 (1.7)	<0.001 ^a^; <0.001 ^b^; 0.133 ^c^
Gross motor ADL	10.4 (2.4)	8.7 (2.2)	8.8 (2.3)	<0.001 ^a^; <0.001 ^b^; 1.00 ^c^
Total ADL	35.1 (6.2)	30.0 (5.2)	28.6 (4.6)	<0.001^a^; <0.001^b^; 0.203 ^c^

ADL = activities of daily living; SD = standard deviation; a = between group 1 and group 2; b = between group 1 and group 3; c = between group 2 and group 3.

**Table 3:** Differences in DCDDaily-Q-ES subscales and total scores for participation and performance in ADL across sex (*n* = 356).

DCDDaily-Q-ES Subscales	Boys *n* = 178 M = 7.33 Years (1.83)	Girls *n* = 178 M = 7.33 Years (1.78)	*p* Value
	Mean (SD)	Mean (SD)	
Participation			
Self-care ADL	15.0 (3.6)	14.6 (3.3)	0.294
Fine motor ADL	9.4 (2.5)	9.3 (2.2)	0.664
Gross motor ADL	12.0 (3.1)	12.7 (2.7)	0.013
Total ADL	36.4 (7.3)	36.7 (6.5)	0.708
Performance			
Self-care ADL	13.4 (3.0)	13.1 (2.8)	0.251
Fine motor ADL	9.3 (2.3)	8.5 (1.9)	<0.001
Gross motor ADL	9.2 (2.4)	9.6 (2.4)	0.095
Total ADL	31.9 (6.4)	31.1 (5.9)	0.261

ADL = activities of daily living; SD = standard deviation.

**Table 4:** Percentiles for participation and performance subscales and total scores across age groups and sex (*n* = 356).

Participation and Performance Subscales	*n*	p80	p85	p90	p95	p96	p97	p98	p99
5–6 years old	136								
Self-care ADL participation		20	21	22	23	24	24	25	32
Fine motor ADL participation		12	13	14	14	15	16	17	18
Gross motor ADL participation		15	16	16	17	17	18	18	19
Total ADL participation		46	47	48	51	52	56	58	67
Self-care ADL performance		17	18	19	20	21	23	24	25
Fine motor ADL performance		12	12	13	14	14	15	15	17
Gross motor ADL performance		12	13	13	14	15	15	15	16
Total ADL performance		40	**41**	43	**45**	47	49	53	56
7–10 years old	220								
Self-care ADL participation		16	16	17	18	19	20	21	21
Fine motor ADL participation		11	12	12	13	13	14	15	15
Gross motor ADL participation		14	15	16	17	17	17	18	20
Total ADL participation		39	41	42	46	47	47	48	50
Self-care ADL performance		14	14	15	16	17	17	19	20
Fine motor ADL performance		10	10	11	12	13	14	14	15
Gross motor ADL performance		11	11	12	13	13	13	15	16
Total ADL performance		33	**34**	36	**39**	40	41	43	45
Boys	178								
Gross motor ADL participation		15	15	16	17	17	18	18	19
Fine motor ADL performance		11	12	13	14	14	15	15	16
Girls	178								
Gross motor ADL participation		15	15	16	17	17	18	18	19
Fine motor ADL performance		10	11	11	13	13	13	14	14

In bold = recommended cutoffs for DCD indication in clinical practice (p85) and research (p95).

**Table 5:** Correlations between DCDDaily-Q-ES performance total and subscale scores and DCDQ-ES total and subscales scores (*n* = 266).

DCDDaily-Q-ES Total and Subscales	DCDQ-ES	Control During Movement	Fine Motor/Handwriting	General Coordination
DCDDaily-Q-ES	0.406 ***	0.340 ***	0.330 ***	0.342 ***
Self-care ADL	0.356 ***	0.311 ***	0.280 ***	0.306 ***
Fine motor ADL	0.307 ***	0.202 ***	0.359 ***	0.255 ***
Gross motor ADL	0.351 ***	0.328 ***	0.199 ***	0.292 ***

Pearson’s correlation coefficients; *** = *p* < 0.001; ADL = activities of daily living.

## Appendix A

**Table A1:** Spanish cut off values and interpretation for the total scores on the “Participation” and “Performance” scales of the DCDDaily-Q-ES.

Participación y Desempeño en AVD	Edades 5 y 6 Años	Edades 7 a 10 Años	Interpretación
Participación en AVD			
Percentil ≥ 95	≥51	≥46	El niño participa mucho menos que sus compañeros en las AVD de acuerdo a la valoración paterna
Percentiles 86–94	48-50	42-45	El niño participa menos que sus compañeros en las AVD de acuerdo a la valoración paterna
Percentil ≤ 85	≤47	≤41	El niño participa lo mismo que sus compañeros en las AVD de acuerdo a la valoración paterna
Desempeño en AVD			
Percentil ≥ 95	≥45	≥39	El desempeño del niño es significativamente peor que el de sus compañeros de acuerdo a la valoración paterna.
Percentiles 86–94	42–44	35–38	El desmepeño del niño es algo peor que el de sus compañeros de acuerdo a la valoración paterna
Percentil ≤ 85	≤41	≤34	El niño no tiene dificultades de desempeño de acuerdo a la valoración paterna

AVD = actividades de la vida diaria.
